# The association between Gabapentin or Pregabalin use and the risk of dementia: an analysis of the National Health Insurance Research Database in Taiwan

**DOI:** 10.3389/fphar.2023.1128601

**Published:** 2023-05-30

**Authors:** Yu-Hua Huang, Mei-Hung Pan, Hwai-I Yang

**Affiliations:** ^1^ Department of Neurology, Chang Gung Memorial Hospital Linkou Medical Center, Taoyuan, Taiwan; ^2^ College of Medicine, Chang-Gung University, Taoyuan, Taiwan; ^3^ College of Medicine, Institute of Clinical Medicine, National Yang-Ming Chiao Tung University, Taipei, Taiwan; ^4^ Genomics Research Center, Academia Sinica, Taipei, Taiwan; ^5^ Graduate Institue of Medicine, College of Medicine, Kaohsiung Medical University, Kaohsiung, Taiwan; ^6^ Biomedical Translation Research Center, Academia Sinica, Taipei, Taiwan

**Keywords:** dementia, Alzheimer disease, cognitive impairment, gabapentin (GBP), pregabalin (PGB)

## Abstract

**Objective:** Previous studies have shown that gabapentin or pregabalin use is associated with cognitive decline. Herein, we aimed to evaluate the association between gabapentin or pregabalin use and the risk of dementia.

**Methods:** In this retrospective, population-based matched cohort study, all research data were collected from the 2005 Longitudinal Health Insurance Database, which contains data of 2 million people randomly selected from the National Health Insurance Research Database of Taiwan in 2005. The study extracted data from 1 January 2000, to 31 December 2017. Adult patients taking gabapentin or pregabalin were included in the exposure group, and patients not using gabapentin or pregabalin matched to exposure subjects in a 1:5 ratio by propensity scores composed of age, sex and index date were included in the non-exposure group.

**Results:** A total of 206,802 patients were enrolled in the study. Of them, 34,467 gabapentin- or pregabalin-exposure and 172,335 non-exposure patients were used for analysis. The mean follow-up day (±standard deviation) after the index date was 1724.76 (±1282.32) and 1881.45 (±1303.69) in the exposure and non-exposure groups, respectively; the incidence rates of dementia were 980.60 and 605.48 per 100,000 person-years, respectively. The multivariate-adjusted hazard ratio of risk of dementia for gabapentin or pregabalin exposure versus the matched non-exposed group was 1.45 (95% confidence interval [CI], 1.36–1.55). The risk of dementia increased with higher cumulative defined daily doses during the follow-up period. Moreover, the stratification analysis revealed that the risk of dementia associated with gabapentin or pregabalin exposure was significant in all age subgroups; however, it was higher in younger patients (age <50) than in the older patients (hazard ratio, 3.16; 95% CI, 2.23–4.47).

**Conclusion:** Patients treated with gabapentin or pregabalin had an increased risk of dementia. Therefore, these drugs should be used with caution, particularly in susceptible individuals.

## 1 Introduction

Dementia is one of the most prevalent neurodegenerative disorders worldwide. It causes progressive impairments in memory, executive function, learning, and ability to perform daily activities (Duong et al., 2017). Multiple factors such as genetics, lifestyle, and environment increase the risk of developing dementia. Researchers are currently investigating risk factors associated with dementia.

An estimated 50 million people live with dementia globally, with over 10 million new cases diagnosed each year (Alzheimer’s Association, 2016). Due to the aging population, the worldwide prevalence of dementia is gradually increasing, especially in North Africa and the Middle East. The number of people suffering from dementia is expected to reach 152 million in 2050 worldwide (Prince et al., 2016; GBD, 2019 Dementia Forecasting Collaborators, 2022). The prevalence of dementia is approximately 1.7%–4.3% among older people (age >65 years), and the number of dementia patients is estimated to reach 303,271 out of a 23 million population in Taiwan on 2022 (Fuh and Wang, 2008; Sun et al., 2014). According to the World Health Organization’s report, people with dementia aged ≥65 years contributed to the population’s disability level more than stroke and cardiovascular disease globally (Lisko et al., 2021). This is significantly linked to medical costs and huge financial burden (Brookmeyer et al., 2007).

As the prevalence of dementia rises, there are increasing concerns regarding gabapentin and pregabalin use owing to their potential contribution to neurocognitive changes. Several publications have reported that these drugs may be associated with cognitive adverse effects (Taipale et al., 2018; Oh et al., 2022). Glutamate and gamma-aminobutyric acid (GABA) are two major neurotransmitters in the central nervous system (CNS). According to previous studies, an impaired glutamatergic system, and alterations of GABAergic circuits in the brain may increase the development of cognitive impairment and Alzheimer’s disease (Li et al., 2016). CNS-affecting drugs, such as benzodiazepines that bind to the GABA receptor have also been associated with the development of dementia (Gray et al., 2016; Gerlach et al., 2022).

Both gabapentin and pregabalin are structural analogs of GABA and can freely cross the blood–brain barrier (Calandre et al., 2016). A study showed that the concentrations of gabapentinoids in the cerebrospinal fluid following oral administration were approximately 9%–14% of the corresponding plasma concentrations (Bockbrader et al., 2010). Gabapentin and pregabalin do not bind to the GABA receptor itself. However, they bind to the alpha*-*2*/*delta-1 subunit of voltage-gated calcium channels on neurons to modulate calcium fluxes, GABAergic neurotransmission and reduce glutamate release (Sills, 2006; Eroglu et al., 2009). This decreases central neuronal excitability, reduces rejuvenating brain plasticity, and blocks the formation of new synapses (Hendrich et al., 2008; Eroglu et al., 2009). It is hypothesized that this attenuation in neuro networking, potentially leading to cognitive adverse effects, especially from the overexpression of α2δ proteins in the hippocampus (Calandre et al., 2016), which plays an essential role in processing declarative and working memories (Yonelinas, 2013).

Initially, the United States Food and Drug Administration (US FDA) and The European Medicines Agency (EMA) had approved gabapentin and pregabalin for neuropathic pain (ex: diabetic peripheral neuropathy, spinal cord injury and post-herpetic neuralgia) and epilepsy adjuvant therapy, especially partial seizures. They are used for similar indications in Taiwan as well. However, the growing trend of gabapentinoid off-label use for various other pain syndromes, alcohol addiction, anxiety, bipolar disorder, and migraines has been noted (Goodman and Brett, 2019). Gabapentin misused or abused alone or with other central nervous system depressants such as opioids, has a risk of respiratory depression, potentially resulting in death (Smith et al., 2016; Evoy et al., 2021).

Other relevant research and systemic reviews have investigated the effects of gabapentinoid drugs on cognitive abilities (Zaccara et al., 2011; Shem et al., 2018; Oh et al., 2022; Oh et al., 2023). However, our study was the first retrospective, population-based cohort study to evaluate the association between gabapentin or pregabalin use and the risk of dementia.

## 2 Material and methods

### 2.1 Data source

This study was based on the data from the National Health Insurance Research Database (NHIRD) in Taiwan. The single-payer National Health Insurance program was initiated in 1995, and 99.9% of the Taiwan’s 23 million population were enrolled. We used the 2005 Longitudinal Health Insurance Database (LHID), which contains data on 2 million people randomly selected from the NHIRD in 2005. Overall, 18 years of data were included, from 1 January 2000, to 31 December 2017. There was no significant difference in the gender, age and average insured payroll-related premiums between the patients in the LHID 2005 and the original NHIRD data (Hsieh et al., 2019). All patient information in the NHIRD was de-identified before being released to researchers*.* The Research Ethics Committee of Academia Sinica approved this study (AS-IRB-BM-18059).

### 2.2 Study design

We used the NHIRD registration, identification, and medical claims files (including inpatient records, ambulatory care records, admission records, and prescription records) for analysis.

Adult patients taking gabapentin or pregabalin were included in the exposure group, and patients not using the drugs were matched to exposure subjects in a 1:5 ratio by propensity scores composed of age, sex and index date were included in the non-exposure group. The index date was defined as the first time pregabalin, or gabapentin treatment was initiated. We extracted data from 1 January 2000, to 31 December 2017, and the index date period was from 1 January 2001, to 31 December 2016. The pre-index period, from 1 January 2000, to 31 December 2000, was used to identify comorbidities and ensure that all study participants had been in the database for at least 1 year. The post-index period, from 1 January 2017, to 31 December 2017, was also included to confirm that all study participants had been followed-up for at least 1 year. The exposure time was defined as 90 days of using gabapentin or pregabalin (Supplementary Figure S1; Study design).

### 2.3 Gabapentin or Pregabalin drug exposure and cumulative defined daily doses (cDDDs).

In clinical practice, the duration of gabapentin or pregabalin therapy mainly depends on the clinical symptoms and adverse effects of the treatment. After a literature review, we were unsure of the duration the drugs would require to cause the side effects of cognitive impairment or dementia. We conducted a preliminary analysis, including gabapentin or pregabalin exposure period of 30 days and 90 days. Lau et al. (1997) reported the validity of pharmacy records in drug exposure assessment. Data on prescription drug use were divided into three different methods, fixed time window of 30 days, fixed time window of 90 days and the calculated duration of use of a prescription All three methods demonstrated high specificity and positive predictive value. The 90-day fixed time window method generally showed high sensitivity (range: 0.67–1.00) than the other two. Based on this data we defined an exposure time of 90 days.

The NHIRD has limited information on medication use; however, the World Health Organization (WHO) recommended the defined daily doses (DDDs) for Drug Statistic analysis. First, we used the formula to quantify the use of gabapentin and pregabalin, as follows: (total exposed dosage)/(amount of drug in DDD) = number of DDDs. We also used cumulative defined daily doses (cDDDs) to measure and standardize exposure to gabapentin and pregabalin for investigating the dose-response relationship between drug exposure and dementia. The hazard ratio (HR) was calculated according to the cDDDs quartile in the subgroup analysis.

### 2.4 Study population and flow chart

In the NHIRD, the international classification of diseases, ninth revision, clinical modification (ICD-9-CM) was used for recording diagnoses before 2015, and the 10th revision (ICD-10-CM) was used for patient diagnoses after 2016. After the literature review, a crosswalk between ICD-9-CM and ICD-10-CM codes were used to identify the comorbidities in our analysis (Quan et al., 2005).

First, we excluded patients who (Duong et al., 2017) were aged ≤20 years and (Alzheimer’s Association, 2016) had at least two primary diagnostic codes or one discharge diagnostic code of epilepsy (ICD-9-CM: 345.xx and ICD-10-CM: G40.x) or herpes encephalitis (ICD-9-CM: 053. xx, and ICD-10-CM: B02) in the hospitalization database.

We further excluded patients who (Duong et al., 2017) were lost to follow-up within 3 months after the index date, (Alzheimer’s Association, 2016), had a dementia diagnosis before the index date or within 3 months after the index date, and (GBD, 2019 Dementia Forecasting Collaborators, 2022) had an index date before 1 January 2001, or after 31 December 2016.

**FIGURE 1:**
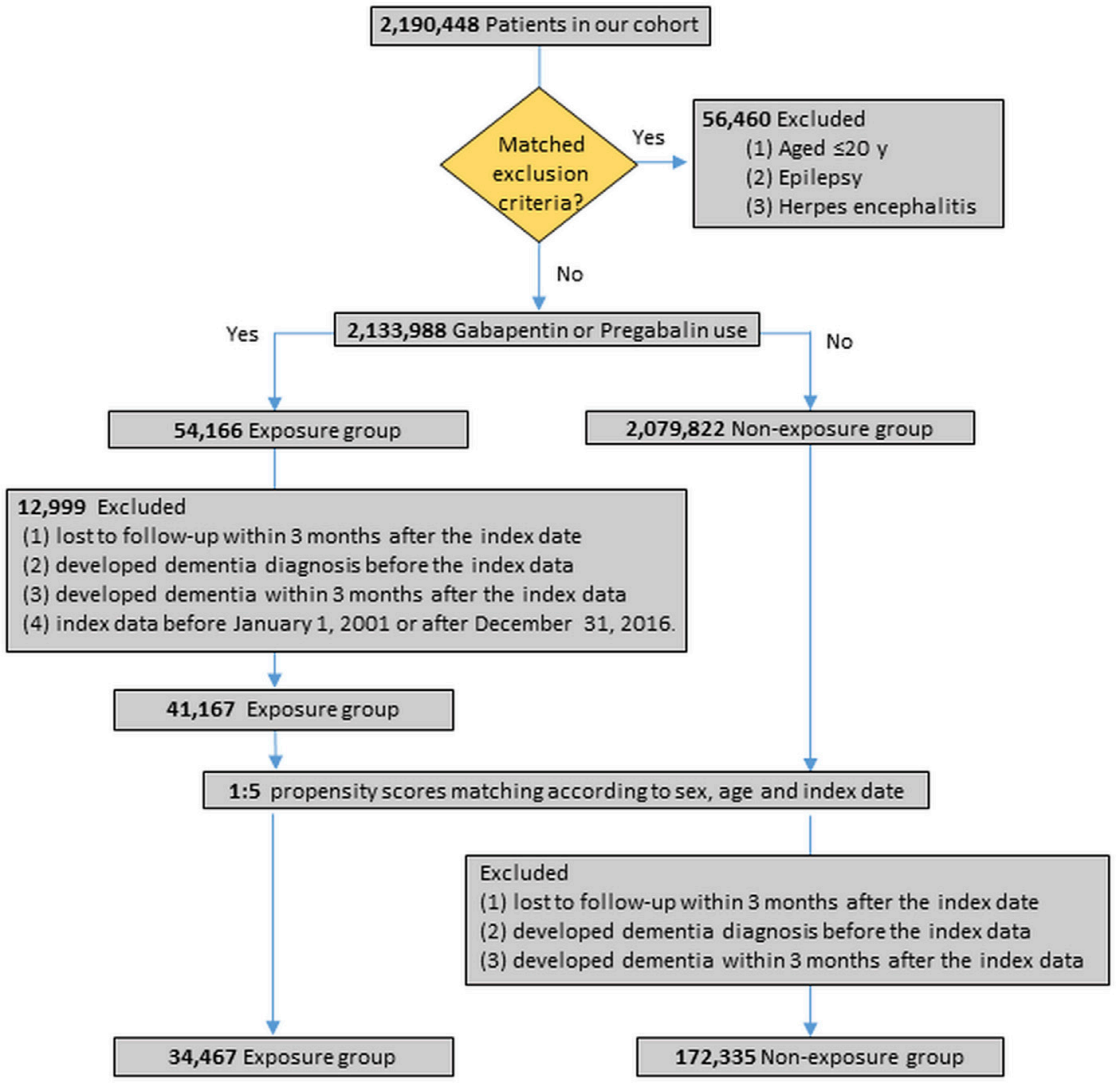
Study flow chart.

As a result, 206,802 patients were enrolled in the analysis, including 34,467 cases and 172,335 controls, respectively. The participants were subsequently divided into two cohorts: pregabalin- and gabapentin-exposed and non-exposed groups (Figure 1. Flow chart).

### 2.5 Outcome

We defined at least two primary diagnostic codes or one hospitalization discharge diagnostic code using the ICD-9-CM code (331.0x, 290. xx, 294.xx) and ICD-10-CM codes (F01, F02, F03, and G30) for dementia. The endpoint of the study was achieved when the patient was diagnosed with dementia or the patient died.

### 2.6 Covariates

Age was categorized into four groups: <50, 50–59, 60–69 and ≧ 70 years for subgroup analysis.

In addition, diabetes mellitus, hypertension, hyperlipidemia, cerebrovascular disease, head injury, and depression have been identified as major risk factors for dementia (Hickman et al., 2016; Edwards et al., 2019; Livingston et al., 2020). Therefore, we identified these risk factors as covariates, which were based on at least two primary diagnostic codes or one hospitalization discharge diagnostic code. Comorbidities were determined using ICD-9-CM and ICD-10-CM codes as follows: diabetes (ICD-9-CM:250. xx; ICD-10-CM: E08∼10, E11, E13), hypertension (ICD-9-CM: 401.xx-405.xx; ICD-10-CM: I10∼I13, I15, N26), stroke (ICD-9-CM: 430–438; ICD-10-CM: G45, G46, I60∼I69), hyperlipidemia (ICD-9-C:272; ICD-10-CM: E71, E75, E77, E78, E88), depression (ICD-9-CM: 296.2x, 296.3x, 300.4x, 311.xx; ICD-10-CM: F32∼ F34), and head injury (ICD-9-CM:800. xx∼804.xx, 850.xx∼854.xx, 959.01; ICD-10-CM: S01, S02, S06, S09). The accuracy of stroke diagnosis in the NHIRD has been validated previously (Cheng et al., 2011). The disease ICD-9-CM and ICD-10-CM codes are listed in Supplementary Table S1.

### 2.7 Statistical analysis

All statistical analyses were performed using SAS version 9.4 (SAS Institute, Cary, NC, United States).

The distributions of patient demographics and comorbidities between the two groups were examined using two-sample *t*-test for the continuous variables, and Pearson’s chi-squared test for the categorical variables. The Kaplan-Meier method was used to estimate the cumulative event rate of dementia, and the log-rank test was used to compare between groups. Cox proportional hazards regression models were used to investigate the associations between exposure to gabapentin or pregabalin and the quartiles of cDDDs of gabapentin or pregabalin exposure and the risk of dementia, adjusting other potential confounders and estimating the hazard ratios (HR) and 95% confidence intervals (CIs). The association between gabapentin or pregabalin exposure and dementia risk was further examined with stratification according to sex, age, and comorbidities such as diabetes, hypertension, stroke, dyslipidemia, depression, and head injury. Statistical significance was set at *p* < 0.05, and all tests were two-tailed.

## 3 Results

This retrospective, population-based cohort study used data from the Longitudinal Health Insurance Database (LHID). The baseline characteristics of the study groups are presented in Table 1. In total, 206,802 patients were enrolled in the analysis, including 34,467 exposure patients and 172,335 non-exposure patients. The follow-up days after the index date (± standard deviation) were 1,724.76 ± 1,282.32 and 1,881.45 ± 1,303.69 in the exposure and non-exposure groups, respectively.

**TABLE 1:** Baseline characteristics, follow-up time and number of incident dementia cases in the Gabapentin or Pregabalin exposed group and matched non-exposed group in our cohort (90 days Gabapentin or Pregabalin exposure time).

		Exposed group	Non-exposed group	*p*-Value
		(n = 34,467)	(n = 172,335)	
Sex	Male	15602 (45.27)	78010 (45.27)	-
	Female	18865 (54.73)	94325 (54.73)	
Age, years (mean ± SD)		54.83 ± 13.93	54.83 ± 13.93	-
Age, years	20–29	1681 (4.88)	8405 (4.88)	-
	30–39	3446 (10)	17230 (10)	
	40–49	6275 (18.21)	31375 (18.21)	
	50–59	9721 (28.2)	48605 (28.2)	
	60–69	8001 (23.21)	40005 (23.21)	
	70–79	4464 (12.95)	22320 (12.95)	
	80–89	857 (2.49)	4285 (2.49)	
	≥90	22 (0.06)	110 (0.06)	
Comorbidities				
	Diabetes mellitus	9490 (27.53)	25265 (14.66)	<0.001
	Hypertension	15605 (45.28)	51921 (30.13)	<0.001
	Stroke	5558 (16.13)	12821 (7.44)	<0.001
	Hyperlipidemia	13062 (37.9)	42244 (24.51)	<0.001
	Depression	2739 (7.95)	5289 (3.07)	<0.001
	Head injury	3354 (9.73)	9910 (5.75)	<0.001
Follow up days (mean ± SD)		1724.76 ± 1282.32	1881.45 ± 1303.69	<0.001
Total years of follow-up		162757.8	887719.9	
Dementia outcome (%)		1596 (4.63)	5375 (3.12)	

Values are presented as the number (percentage) or mean ± SD.

Statistical significance was considered as *p*-value <0.05.

The two-sample *t*-test or Chi-square test was used for continuous variables and categorical variables, respectively.

A total of 1,596 and 5,375 dementia cases newly developed during 162,757.8 and 887,719.9 years of follow-up in the exposure and non-exposure groups, respectively, accounting for the incidence rate of dementia of 980.60 and 605.48 per 100,000 person-years, respectively. The HR (95% CI) of dementia for gabapentin or pregabalin exposure was 1.45 (1.36–1.55) compared to non-exposure group, after adjustment for diabetes mellitus, hypertension, stroke, hyperlipidemia, depression, and head injury.

**FIGURE 2:**
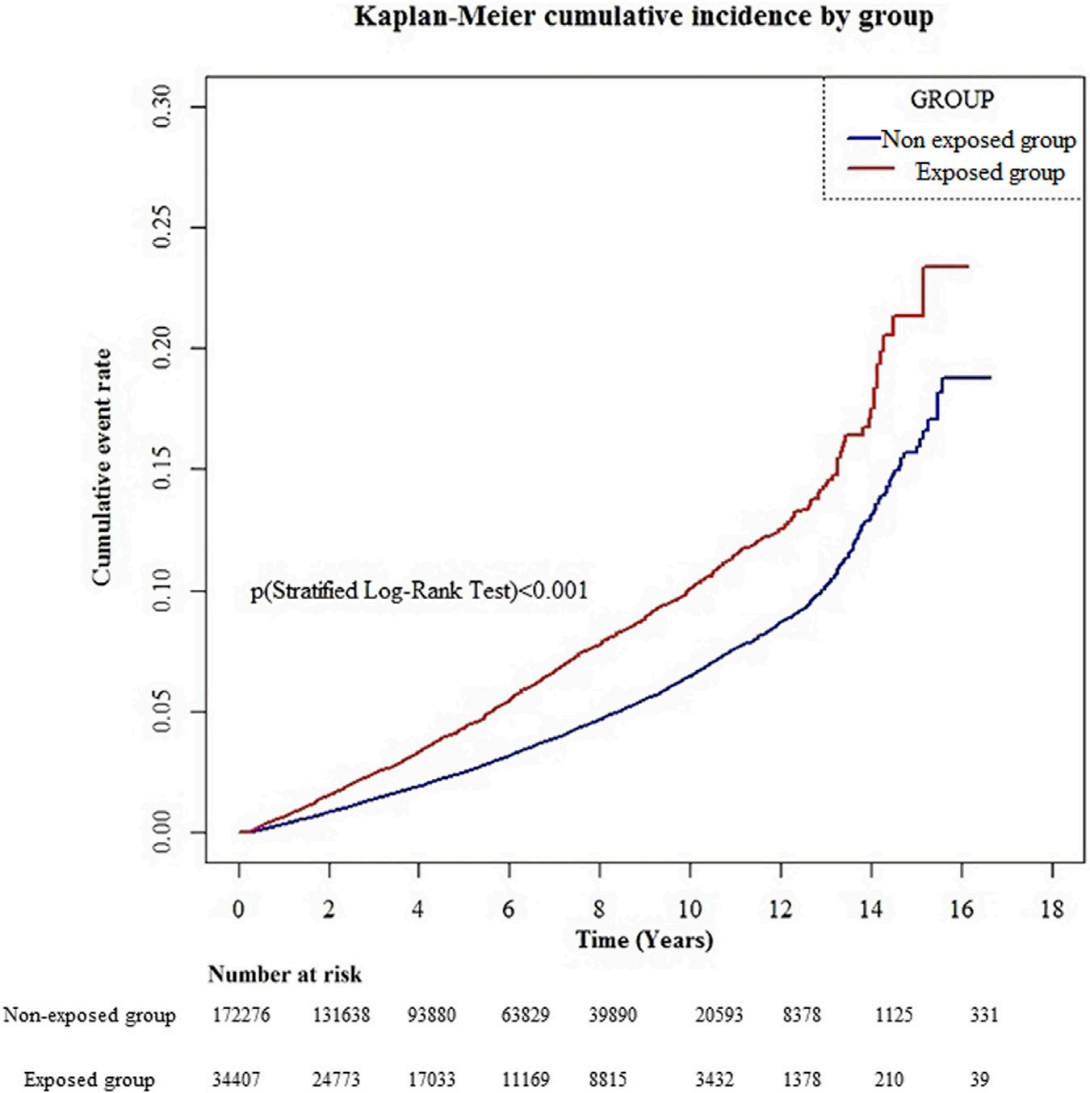
Cumulative event rate of dementia.

The cumulative incidence curve revealed that the gabapentin- or pregabalin-exposed group had a significantly higher cumulative incidence of dementia than the non-exposed group during the entire follow-up period (Figure 2; log-rank test *p* < .001).

Among gabapentin or pregabalin exposure group, the mean (±SD) cDDDs per year during follow-up was 28.12 (±139.54); median (Q1-Q3) was 1.95 (0.50–9.66). We further investigated the association between the quartiles of cDDDs of gabapentin or pregabalin exposure and the risk of dementia and discovered that HRs increased with increasing cDDDs per year during follow-up. The comorbidities-adjusted HR was 1.24 (95% CI, 1.09–1.41; *p* = 0.001), 1.69 (95% CI, 1.50–1.92; *p* < 0.001) and 2.44 (95% CI, 2.14–2.78; *p* < 0.001) for cDDDs 0.50–1.95, 1.96–9.66 and >9.66, respectively, compared to cDDDs <0.5 as the referent (Table 2).

**TABLE 2:** Risk of dementia increased with increasing Cumulative Defined Daily Doses (cDDDs) per year during follow-up.

Variable		HR (95% CI)	*p*-value^†^
		Adjusted	
Gabapentin or pregabalin cDDDs per year during follow-up			
mean ± SD	28.12 ± 139.54		
median (Q1–Q3)	1.95 (0.50–9.66)		
<0.50	8616 (25)	1	
0.50–1.95	8622 (25.02)	1.24 (1.09–1.41)	0.001
1.96–9.66	8613 (24.99)	1.69 (1.50–1.92)	<0.001
>9.66	8616 (25)	2.44 (2.14–2.78)	<0.001

Cox regression (event: dementia outcome or death; time variable: time from onset to dementia outcome or death).

^†^ Adjusted for diabetes mellitus, hypertension, stroke, hyperlipidemia, depression, and head injury.

**FIGURE 3:**
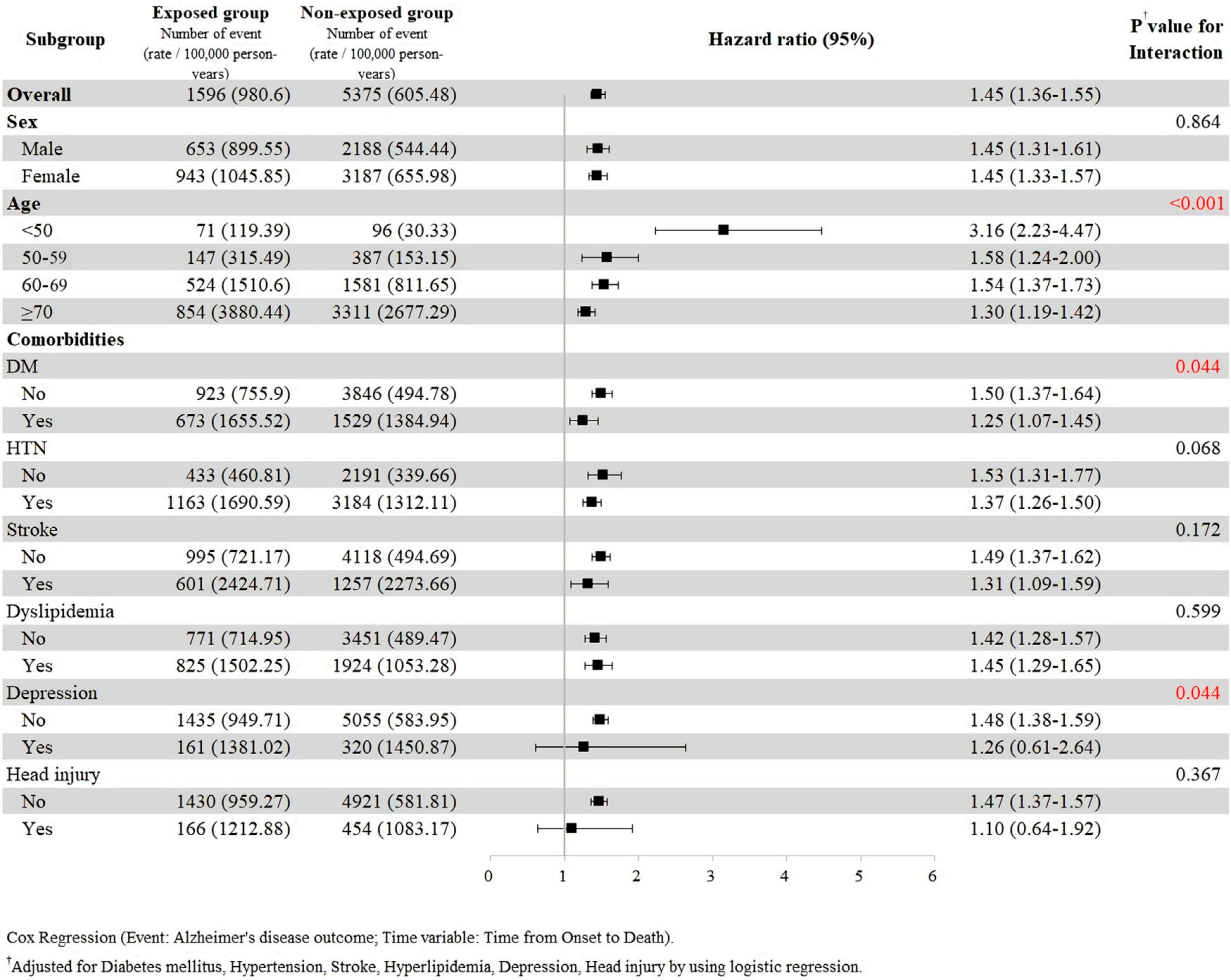
Primary outcome in subgroups.

The subgroup analysis with stratification according to sex, age, and comorbidities was further performed (Figure 3). The results revealed that the risk of dementia associated with gabapentin or pregabalin exposure was significant in all subgroups except for the strata having depression or head injury. The risk of dementia development was higher in the younger group (age <50 years) than that in the older group. The comorbidities-adjusted HR was 3.16 (95% CI, 2.23–4.47) in the age group <50, 1.58 (95% CI, 1.24–2.00) in the age group 50–59, 1.54 (95% CI, 1.37–1.73) in the age group 60–69, and 1.30 (95% CI, 1.19–1.42) in the age groups ≥70 years.

## 4 Discussion

Memory impairment and cognitive decline are two of the greatest concerns with long-term administration of drugs that affect the CNS. In this study, we discovered a significant association between cumulative exposure to gabapentin and pregabalin and the risk of dementia.

Particularly, individuals <50 years and those with higher accumulative dose were more susceptible.

The results from our study are consistent with previous studies. An association has been considered possible between gabapentin use and cognitive decline in individuals with spinal cord injury (Shem et al., 2018). However, this study had a small sample size and short follow-up duration without a control group (Shem et al., 2018). A cross-sectional study, including 300 patients with pregabalin misuse and 100 controls, showed greater cognitive impairment in the patient group than in the control group (*p* < .001) (Mohamed and Emam, 2020). In this study, no association was determined between the dose of pregabalin and cognitive impairment (Mohamed and Emam, 2020). In a retrospective cohort study using the National Alzheimer’s Coordinating Center Uniform Data Set, gabapentin initiation in cognitively normal older adult research volunteers (age ≥65 years; 480 initiators; 4,320 nonusers) was significantly associated with deleterious neurocognitive changes in the 2 years after initiation (Oh et al., 2022). However, in this study, even in investigations that included healthy volunteers, the study period was short (only a few weeks) and the doses of gabapentin use were limited.

In a review of post-marketing surveillance of pregabalin and gabapentin, most patients generally tolerated these two drugs well during treatment. Approximately 4% of patients discontinued treatment due to adverse effects. For both drugs, the most frequently reported neuropsychiatric symptoms were dizziness, somnolence, fatigue and confusion (Quintero, 2017). Hallucinations, agitation, and aggressiveness have also been highlighted (Quintero, 2017). Approximately 29.1% of gabapentin users and 35.2% of pregabalin users had these neuropsychiatric adverse effects (Fuzier et al., 2013). Adverse reactions of both drugs were mainly mild-to-moderate, generally dose*-*dependent, and transient in nature after dose reduction (Bockbrader et al., 2010). Recent studies, including meta-analyses, have focused on the neuropsychiatric adverse effects induced by these two drugs (Ho et al., 2006; Hurley et al., 2006; Zaccara et al., 2011; Wiffen et al., 2017; Derry et al., 2019).

FDA-approved indications for gabapentin and pregabalin are seizure and neuropathic pain; however, off-label use for anxiety, non-neuropathic pain, mood instability, and alcohol withdrawal symptoms has gradually increased (Bonnet and Scherbaum, 2017). Gabapentinoid prescriptions have increased in the United States (Johansen, 2018), the United Kingdom (Montastruc et al., 2018) and Europe (Persheim et al., 2013; Priez-Barallon et al., 2014). These medications have the potential for misuse, addiction, and overdose, when combined with opioids or benzodiazepines. Both gabapentin and pregabalin are structural analogs of GABA. They do not bind to the GABA receptor itself. However, they bind to the alpha*-*2*/*delta-1 subunit of voltage-gated calcium channels on neurons to modulate calcium fluxes, GABAergic neurotransmission and glutamate release at nerve terminals (Sills, 2006; Eroglu et al., 2009) Glutamatergic and GABAergic neurotransmitters are the two major types of neurotransmitters in the central nervous system (CNS). These drugs not only block the development of hyperalgesia and central sensitization, but also inhibit the release of excitatory neurotransmitter, including glutamate, norepinephrine (noradrenaline), serotonin, and dopamine (Hendrich et al., 2008; Eroglu et al., 2009). The dopamine reward system may play a role in gabapentinoid abuse and addiction (Althobaiti et al., 2021). The hippocampus, with abundant alpha*-*2*/*delta-1 subunit of voltage-gated calcium channels, plays an essential role in processing declarative memories and working memory (Yonelinas, 2013). It is hypothesized that this attenuation in neuro networking, potentially leading to cognitive adverse effects (Calandre et al., 2016).

Some patients use gabapentinoids as antiepileptic drugs. Gabapentinoids are anticonvulsants that reduce synaptic transmission by decreasing presynaptic voltage-gated Ca2+ and Na + channels (Lasoń et al., 2013). In a Finnish and German analysis of healthcare registers and insurance datasets, regular use of antiepileptic drugs (AEDs), including gabapentin and pregabalin, demonstrated an increase in cognitive impairment and dementia risk (Taipale et al., 2018). Several studies suggest that using gabapentin and pregabalin for epilepsy control is associated with an increased risk of dementia, and the effect appears to be lifelong (Knight et al., 2021). However, these studies specifically involved patients with epilepsy or medically ill patients. In previous reviews, approximately 48% of patients with epilepsy had cognitive impairments and memory problems (Guekht et al., 2007). Many factors, including epilepsy type, attack duration, etiology, and severity of seizure, can contribute to these results, which are unrelated to gabapentinoids (Park and Kwon, 2008; Eddy et al., 2011). In addition, encephalitis, especially herpes encephalitis, has been reported to cause disability, cognitive deficits, and intractable epilepsy (Noppeney et al., 2007; Michaeli et al., 2014). Thus, we focused on the association between gabapentinoids and dementia risk. In our studies, we first excluded patients with diagnoses of seizure and encephalitis.

Especially in older adults, gabapentin and pregabalin are prescribed to treat behavioral and psychological symptoms of dementia (BPSD). A systematic review analyzing 24 relevant articles found that the use of gabapentinoid agents significantly decreased BPSD in patients with Alzheimer’s disease, suggesting a possible benefit. However, 15 papers were original case series/case reports, and the remaining 9 papers were solely reviews. There were no randomized trials.

In our analysis, the possibility of dementia development associated with gabapentin or pregabalin exposure appeared to be higher in the younger group compared with the older group. This finding is a true novelty of this article. Gabapentinoid agents are absorbed gastrointestinally via the l-amino acid transport system in the proximal small bowel (Berry et al., 2003). Gabapentinoids have large inter-individual pharmacokinetic variability due to saturable absorption and variable renal function of the patients (Yamamoto et al., 2022). A possible explanation for impact of age in our study could relate to older adults having more polypharmacy, higher co-morbidities, and decreased renal function, all of which interfere with the absorption of gabapentinoids. Further studies should consider gabapentinoid dose bioavailability and dose-serum concentration analysis between different age groups to evaluate the possible etiology of this relationship. However, recent studies corroborated evidence that female sex and patients aged <35 years lent to a higher likelihood of gabapentinoid abuse and addiction (Evoy et al., 2021). In our study, the subgroup analysis revealed that younger patients were more susceptible to develop dementia. Combining both issues with the potential for drug dependency and cognitive function impairment, we should use these drugs with caution in younger patients. We also need to be cautious in younger patients with a history of substance abuse, particularly that involving benzodiazepines and opioids.

Our study has several strengths. First, our cohort studies had a large sample size, with 34,467 and 172,335 patients in the exposure and non-exposure group, respectively. Second, the follow-up time after the index date was long, at nearly 5 years (1724.76 ± 1282.32 in the exposure group and 1881.45 ± 1303.69 in the non-exposure groups).

The study also has some limitations. First, as in many previous NHIRD-based studies, it was retrospective in nature and relied on the ICD-9-CM and ICD-10-CM codes instead of direct medical records or interview data. Therefore, errors related to lack of detailed documentation and misdiagnosis may have occurred. Second, neuropsychological tests, including the mini-mental state examination, clinical dementia rating scale, and cognitive abilities screening instrument, were not provided in the NHIRD. Therefore, detailed information on the severity of dementia and its clinical staging was unavailable. Third, we used cDDDs of gabapentinoids in this study, which only assumed the average maintenance dose per day. Fourth, the NHIRD lacked patients’ lifestyle information (such as, smoking status and alcohol consumption), which may have affected the incidence of dementia. Fifth, we had limitations in eliminating the impacts of different dementia types. Sixth, we only examined the adverse effects of gabapentinoids. We did not estimate the effect of the concomitant medications; confounders such as benzodiazepines, antihistamines, anticholinergics/antimuscarinics, tricyclic antidepressants (TCAs), muscle relaxants, opioids, proton pump inhibitors, antiepileptic drugs, antiparkinson drugs, and antipsychotics. Finally, it was likely that residual confounding effects could still exist due to those unmeasured variables, including chronic pain conditions, mood and anxiety disorders (other than depression) and psychotic disorders. In a longitudinal, population-based cohort study, chronic pain was associated with accelerated memory decline (Whitlock et al., 2017). In 2016, Petkus et al. (2016) reported that anxiety symptoms were also associated dementia development. In the literature review, people with schizophrenia have a nearly twofold to threefold increased risk of dementia after adjusting for other standard risk factors (Cai and Huang, 2018; Lin et al., 2018; Almeida et al., 2019). Future studies should analyze the concomitant medications and these additional comorbid conditions as confounders.

Dementia is a slow and progressive neurodegenerative disorder. Although in our cohort, a study period of 18 years was sufficient to observe the association between gabapentinoid use and dementia. This finding deserves further validation in cohort studies with longer follow-up periods.

## 5 Conclusion

In conclusion, in this analysis of the NHIRD in Taiwan, we observed an association between gabapentin and pregabalin use and dementia risk. Apart from the well-described neuropsychiatric effects associated with gabapentinoids, cognitive impairment and dementia should be considered, especially in long-term treatment, patients with higher cDDDs, and younger patients. However, our study was a retrospective NHIRD cohort study. Therefore, further prospective investigations are required to understand the mechanism of dementia development with these two widely used drugs in the future.

## Data Availability

The datasets presented in this article are not readily available because This study was based on the data from the National Health Insurance Research Database (NHIRD) in Taiwan. Taiwan’s Ministry of Health and Welfare (MOHW) established a Data Center for on-site data analyses. Several policies and analytical approaches have been developed to ensure the privacy and safety of data. Requests to access the datasets should be directed to Yuhua Huang, yuhua0126@gmail.com.
