# Supplementary material for: The association between Gabapentin or Pregabalin use and the risk of dementia: an analysis of the National Health Insurance Research Database in Taiwan
Source: Front Pharmacol. 2023 May 30;14:1128601. doi: 10.3389/fphar.2023.1128601 (PMC10266423; doi:10.3389/fphar.2023.1128601)
Supplement: Supplementary file 1 [file Table1.DOCX]

**Supplementary table 1**. The crosswalk table of the disease ICD-9-CM and ICD-10-CM codes

| Diseases | ICD-9-CM code | ICD-10-CM code |
| --- | --- | --- |
| Dementia | 331.0x, 290.xx, 294.xx | F01, F02, F03, G30 |
| Encephalitis | 053.xx | B02 |
| Epilepsy | 345.xx | G40.x |
| Diabetes mellitus, DM | 250.xx | E08, E09, E10, E11, E13 |
| Hypertension, HTN | 401.xx~405.xx | I10~13, I15, N26 |
| Stroke | 430~438 | G45, G46, I60~I69 |
| Hyperlipidemia | 272 | E71, E75, E77, E78, E88 |
| Depression | 296.2x, 296.3x, 300.4x, 311.xx | F32, F33, F34 |
| Head injury | 800.xx~804.xx, 850.xx~854.xx, 959.01 | S01, S02, S06, S09 |

ICD-9-CM, International Classiﬁcation of Disease, Ninth Revision, Clinical Modiﬁcation; ICD-10-CM, International Classiﬁcation of Disease, Tenth Revision, Clinical Modiﬁcation.
