# Supplementary figures and images for: The association between Gabapentin or Pregabalin use and the risk of dementia: an analysis of the National Health Insurance Research Database in Taiwan
Source: Front Pharmacol. 2023 May 30;14:1128601. doi: 10.3389/fphar.2023.1128601 (PMC10266423; doi:10.3389/fphar.2023.1128601)

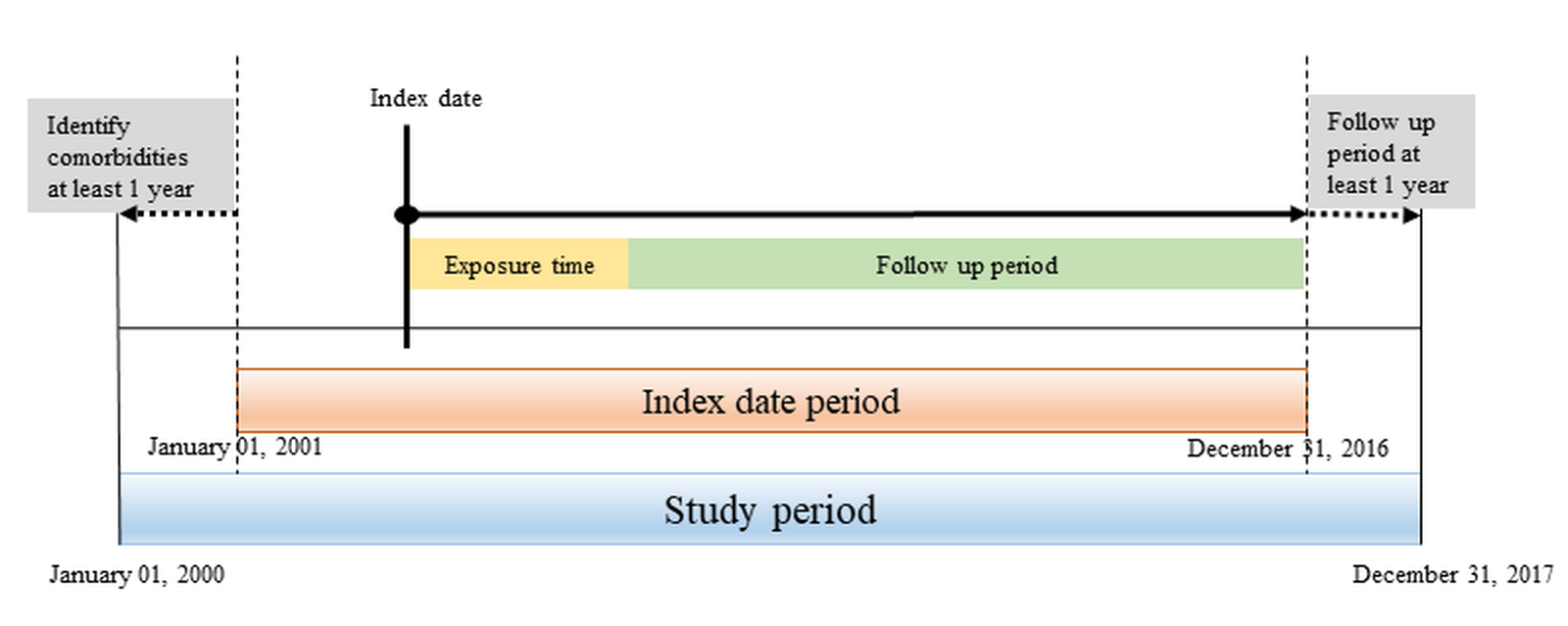

Supplement: Supplementary file 2 [file Image1.TIF]
